# From complexity to parsimony: A systems thinking validation of the multiple streams framework in abortion policy agenda setting

**DOI:** 10.1186/s12961-025-01392-y

**Published:** 2025-10-28

**Authors:** Gabriela Alvarado

**Affiliations:** RAND Corporation, 1200 South Hayes St, Arlington, VA 22202-5050 USA

## Abstract

**Background:**

Abortion policy is a highly contested area of health policy. Despite international recognition of abortion as a human right, legal restrictions persist in many countries, and recent decades have seen both liberalization and retrenchment of abortion laws. While much research has examined the politics and outcomes of abortion policy reform, less attention has been paid to the upstream process of how abortion emerges on the policy agenda. This study addresses this gap by focusing on agenda setting for abortion policy, using Kingdon’s multiple streams framework (MSF) and systems thinking.

**Methods:**

This exploratory study integrates MSF with systems thinking and causal loop diagramming to map the dynamic interactions among government characteristics, policy communities, policy-maker attributes and external events. A purposive review of 19 key works from the MSF literature was conducted, selected for their theoretical contributions and detailed descriptions of stream interactions. Qualitative text coding and quotation analysis were used to identify causal relationships, which were then aggregated into a causal loop diagram. Model validation focused on micro-structure elements, and the framework was tested against two case studies: Ireland and Nicaragua.

**Results:**

Analysis yielded 167 unique elements and 338 causal links, distilled into 81 key variables. The causal loop diagram demonstrates that convergence of the problem, policy and politics streams is shaped by reinforcing and balancing feedbacks, rather than random chance. Key factors influencing agenda setting include party institutionalization, policy entrepreneur effectiveness, social inequality and the gravity of focusing events. The case studies illustrate how variations in political institutions, mobilization efforts and external events can lead to divergent policy trajectories.

**Conclusions:**

This study provides theoretical validation that the parsimonious MSF can account for the complexity of abortion policy agenda setting when integrated with systems thinking. The causal loop diagram identifies actionable leverage points for advocacy and policy reform and offers a dynamic, testable model for understanding agenda setting in contentious policy domains. These findings bridge theoretical innovation with practical relevance, laying a foundation for future empirical research and offering insights for scholars, advocates and decision-makers seeking to influence the policy agenda for abortion and other complex health issues.

## Background

Abortion is a highly contested area of health policy given the complex interplay of religion, culture, public health, human rights and social equity. Despite the recognition of access to abortion as a human right in numerous international conventions and treaties, legal restrictions to abortion persist in many countries around the world [1]. For most of the nineteenth and the first half of the twentieth century abortion was illegal in most countries; however, in recent decades countries across the globe have experienced both liberalization and retrenchment of abortion laws [2, 3]. Understanding not only why abortion policy reform occurs but how abortion even gets on to the policy agenda is a challenge for researchers, advocates and policy-makers.

A substantial body of literature across numerous academic disciplines have examined the politics of abortion at the country level, including the roles of religious institutions, social movements, political parties and international influences in shaping abortion policy reform [3–5]. Nonetheless, the focus of these studies is often centred around the content of policy debates, the outcomes of judicial and legislative processes or the public health impact of increased access to abortion, rather than the upstream question of how abortion can emerge as an issue that is thought to require a policy action in the first place.

This study addresses this gap by focusing specifically on the process of agenda setting for abortion policy. Theories of agenda setting are fitting in this situation, as they aim to articulate why some issues rise to the forefront and capture the attention of the public and policy-makers, while others are forgotten [6, 7].

**Fig. 1:**
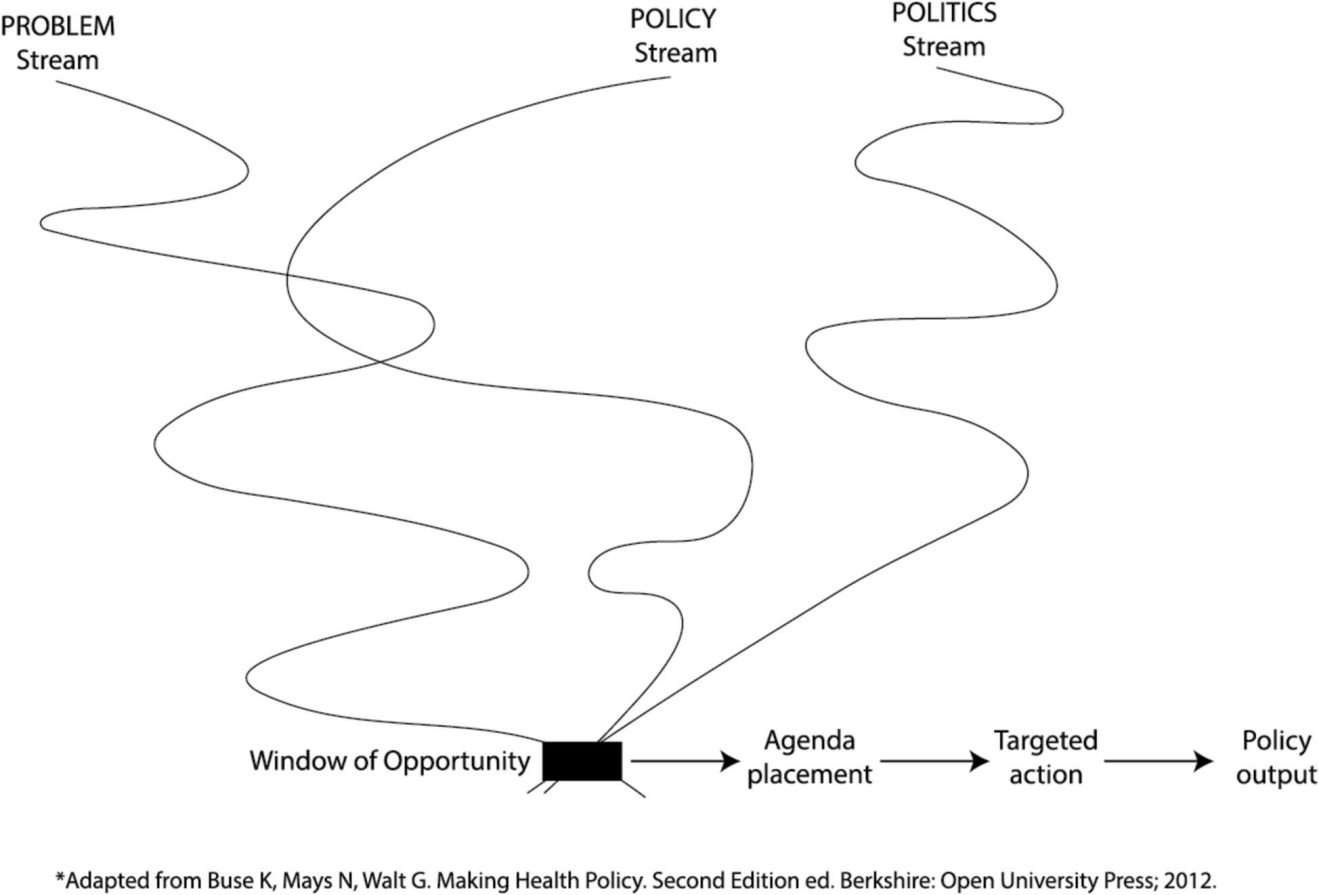
Kingdon’s multiple streams framework [9]

One useful theoretical framework to contextualize agenda setting was described by John Kingdon in 1984 [8]. Kingdon’s multiple streams framework (MSF) proposes that there are three parallel streams – problems, politics and policy – and when these three streams come together, a “window of opportunity” is created where the timing is optimal for placing the issue on the policy agenda (Fig. 1 [9]). The problem stream refers to the goal gap – meaning the difference we can observe between the current state and the desired state. However, there is no universal problem definition, and many people may have different perceptions and definitions for the same problem. The policy stream represents the universe of possible solutions and alternatives to address the problem. Lastly, the politics stream refers to the mood of the people and whether the public and/or elected officials care about the problem as stated. When the three streams come together, this window of opportunity represents an optimal timing of issue placement on the policy agenda which can increase likelihood of a targeted action and thus a favourable policy outcome. For abortion policy reform, where the path to change is rarely linear, the MSF provides a helpful approach to examine the conditions under which abortion can break and enter on to the policy agenda.

Models are increasingly accepted strategies to examine complex occurrences [10], as is evidenced by Kingdon’s multiple streams framework being used broadly across the political science literature. While MSF has been widely applied to health policy and beyond, its application to abortion policy has often been limited to descriptive accounts, with less attention to the underlying system dynamics that shape the emergence and closure of policy windows. At the same time, systems thinking has gained traction in health policy research as a means to understand complex, adaptive systems and to identify leverage points for intervention [11, 12]. Causal loop diagrams are a core tool of systems thinking which allow researchers to visualize the interconnections and feedback loops among system elements, offering a dynamic perspective on policy processes [13].

In this study, I bring together Kingdon’s MSF with a systems thinking approach to advance understanding of how abortion policy gets onto the policy agenda. By systematically mapping the causal relationships and feedback loops among key variables identified in the MSF literature, I aim to move beyond static models of policy change. The aim of integrating systems thinking with MSF is to clarify the underlying dynamics within the MSF to have the tools to make better predictions regarding future states [11].

This approach provides a more nuanced account of how government characteristics, policy communities, policy-maker attributes and external events interact to shape the likelihood that abortion will become a priority for policy action. Part of the theoretical underpinnings of the MSF posit that the three streams sometimes move and change in “random” ways. By carefully mapping out the dynamics between the streams we can elucidate the interactions within variables and streams – thus avoiding the notion that the system cannot be controlled.

This work makes two primary contributions. First, it offers a novel synthesis of agenda-setting theory and systems thinking, demonstrating how causal loop diagramming can enrich the analysis of policy windows and advocacy strategies. Second, it provides actionable insights for those seeking to advance or defend abortion rights, by identifying critical leverage points within the policy system. Through two illustrative case studies, I show how this integrated framework can help explain divergent policy trajectories Ireland and Nicaragua.

By focusing explicitly on the agenda-setting stage of abortion policy and bridging theoretical innovation with practical relevance, this study seeks to inform both the scholarly literature on health policy change and the strategies of advocates and decision-makers working in the field of abortion policy.

## Methods

### Study design and approach

This exploratory study employs a systems thinking approach to examine how abortion policy emerges on the policy agenda, with a particular focus on testing the adequacy of Kingdon’s multiple streams framework (MSF) for capturing the complexity of this process. Systems thinking is well-suited to analysing chronic, multifaceted policy challenges such as abortion reform, where multiple actors, institutions and contextual factors interact over time and where previous efforts to understand or influence change have often fallen short [12, 14, 15].

Systems thinking involves describing the purpose of the system and the key elements and mapping the interconnections and how these generate patterns of behaviour over time [12, 14]. In the context of agenda setting for abortion, this means we can explore the dynamic feedbacks and leverage points that shape whether, how and when abortion becomes a priority for policy action.

To operationalize this approach, I use causal loop diagrams which are particularly valuable for identifying reinforcing and balancing processes and clarifying how changes in one part of the system can ripple through to affect overall outcomes [11]. This method allows for a transparent and systematic assessment of whether the streamlined structure of MSF can realistically account for the diverse and complex drivers of abortion policy agenda setting.

In this study, abortion policy reform is conceptualized as a chronic issue within the broader system of health policy agenda setting. By integrating MSF with systems thinking and causal loop diagramming, this study aims to provide both a theoretical validation of MSF’s applicability and practical insights for advocates and policy-makers.

### Data sources

To examine the fit of the MSF in capturing the complexity of abortion policy agenda setting, I conducted a purposive review of the MSF literature. Rather than a systematic review, I selected 19 works that represent major theoretical contributions and provide enough detail to disaggregate the MSF components. I chose this approach to ensure coverage of foundational and innovative perspectives, as well as allowing for in-depth analysis of the MSF framework.

I identified sources through targeted searches on PubMed and Web of Science databases, manual reference mining and the “cited by” and “related articles” tools on Google Scholar. Additionally, I conducted targeted literature reviews on specific areas and phenomena related to Kingdon’s multiple streams framework that needed further clarification. The inclusion criteria for the articles were any of the following: advanced or refined MSF theory, provided detailed descriptions of the streams and their interactions, or were frequently cited in the policy literature. I assessed for saturation by keeping a log of new variables across reviewed articles. When no new elements were identified for three consecutive articles, I considered the sample to be sufficient. The full list of included references and a summary of each article is provided in the Appendix.

### Purposive text analysis and causal mapping

To systematically map the variables and interactions within the MSF, I adapted purposive text analysis methods to produce a causal loop diagram [13, 16]. The process involved the following steps:

1. Qualitative text coding to select data segments
2. Rigorously interpreted quotation analysis
3. Representation of causal chains in a simple diagram
4. Causal loop diagram development
5. Model validation

#### Qualitative text coding to select data segments

I conducted an initial review of the selected articles and then proceeded to a round of open coding to help refine the problem definition, system boundaries and preliminary information on the dynamics of the multiple streams framework [17]. I used this initial round of open coding to develop definitions and boundaries for a simple codebook to code all included articles (Table 1). The second round of coding focused on selecting data segments that contained causal language with an argument and supporting rationales [16]. Qualitative coding was conducted with the assistance of Dedoose software (version 9.0.82).^1^

**Table 1:** Codebook used for qualitative coding

Code	Definition
1. Model background	General information about Kingdon’s multiple streams framework, relevance in the field, how it is/can be used and the evolution of the model over time
2. Problem stream	How problems are defined and come to capture the attention of the public and decision-makers. Includes: - Focusing events - Political pressure - Indicators and metrics - Magnitude of problem - Public visibility of issue - Emergence of powerful symbols - Budget - Values alignment
3. Policy stream	Whether there are any feasible solutions to a given problem. Includes: - Community of specialists - Degree of fragmentation within the community of specialists - Existence of a common language - Technical feasibility - Available alternatives
4. Politics stream	What the political landscape surrounding a policy issue looks like. Includes: - Public mood - Pressure group campaigns - Elections - Partisan distribution - Media coverage - Political mobilization - Political elites - Coalition building
5. Policy entrepreneur	Advocates for a particular issue who have a willingness to invest time and resources into the issue. Includes: - Incentives for advocacy - Sense of duty - Claim to hearing - Political connections - Negotiating skills - Persistence
6. Window of opportunity	Descriptions of “the right moment” for action. Includes: - Connecting a problem to a policy solution - Decision agenda - Priority-setting - Duration of focusing event - Opportunity cost - Budget cycles - Spill over from other policy issues

#### Rigorously interpreted quotation analysis

**Fig. 2:**
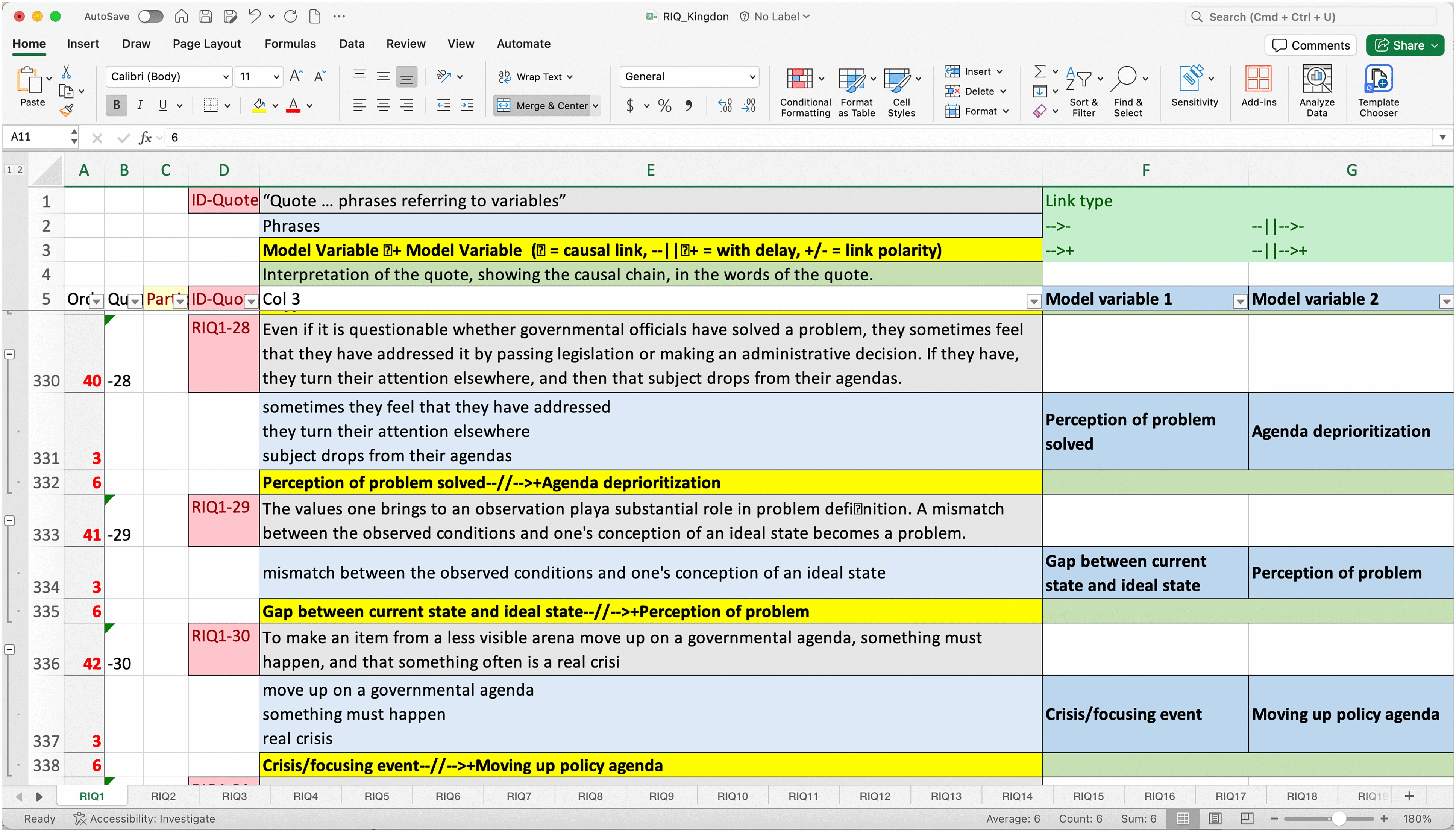
Example of rigorously interpreted quotation analysis to identify individual elements within the system, as well as causal chains and relationships between elements

Once the coding was finalized, I exported all the relevant coded excerpts to Excel. I constructed causal chains of events using rigorously interpreted quotation analysis with the coded excerpts [18]. This involves identifying the cause-and-effect variables in each data segment and polarity of the relationship. Figure 2 is a screenshot of how each individual excerpt was analysed. For example, in excerpt RIQ1-28 in the figure, I identified the key phrases and wrote them down in the light blue box, then identified the specific elements or variables and listed those in the dark blue boxes and lastly added a relationship (either “+"or “−”), which constructed the draft causal chain seen in the yellow box.

#### Representation of causal chains in a simple diagram

Once the rigorously interpreted quotation was finalized, I iterated on the elements and variables to aggregate repeated concepts and simplify as much as possible, while still keeping enough detail to depict realistic dynamics within the system. I then took the final list of causal links and manually drew a map of how these may fit together.

#### Causal loop diagram development

After the simple draft diagram was completed, I proceeded to refine arguments, elements and relationships and aggregated into a causal loop diagram using Kumu,^2^ an online tool designed to visualize data maps and complex relationships.

Causal loop diagrams consist of a very simple set of structural elements: variables and arrows. Variables are all the components within the system, and they must be named or labelled with a direction in terms of quantity or degree. Arrows are used to connect the variables to indicate a relationship of cause and effect, all arrows were notated with a positive (+) or negative (−) polarity, which indicates the nature of the relationship between the two elements. Additionally, arrows can have a double strikethrough line, which indicates there is a time delay between the cause and effect [13].

Key considerations I kept in mind as I either expanded or pruned the causal loop diagram were the core problem I was attempting to address, which concepts and variables needed to remain endogenous to the system and what drove the described behaviours of the included variables.

#### Model validation

The final step in my process consisted of a validation process. The validity of a model is the extent to which it appropriately represents the system in question, which in turn impacts the model’s usefulness and effectiveness [19]. Groesser and Schwaninger describe a hierarchy of validation which starts at the base with element validation and escalates through simple dynamics, multiple dynamics, full dynamics and meta-level validation [19]. This hierarchy mirrors the levels of complexity of the model, and as such, the escalation to the next level of validation requires more intensive resources and time.

Given the exploratory nature of this work and the limited prior research applying systems thinking to the MSF in the context of abortion policy agenda setting, validation efforts were intentionally concentrated at the micro-structure level of the model: the element level and simple dynamics. This approach is well-suited for early-stage conceptual modelling, allowing for a rigorous assessment of whether the identified elements and relationships accurately represent the system and generate plausible dynamics, thereby laying a strong foundation for future, more comprehensive validation efforts. (Table 2) [19].

**Table 2:** Micro-structure causal loop diagram validation approach

Validation hierarchy level	Test	Detail
Element level	Parameter verification	Ensure that elements in the model represent an actual element of the system under study.
	Structure verification	Verify that the relationships depicted in the model really exist in the system under study.
	Dimensional consistency	Make sure that the elements in the model are dimensionally consistent.
Simple dynamics	Indirect extreme condition	Test the feasibility of the feedback loops by setting parameters at extreme levels.
	Integration error	Check whether the results are independent of the integration time constant.
	Behaviour sensitivity^*^	Focus on the sensitivity of the model and whether it matches the expected behaviour of the real system.
	Boundary adequacy^*^	Determine whether the elements within the model structure suffice to generate realistic dynamics.

^*^Meso-structure level tests that will be included in the causal loop diagram’s validation efforts

## Results

**Fig. 3:**
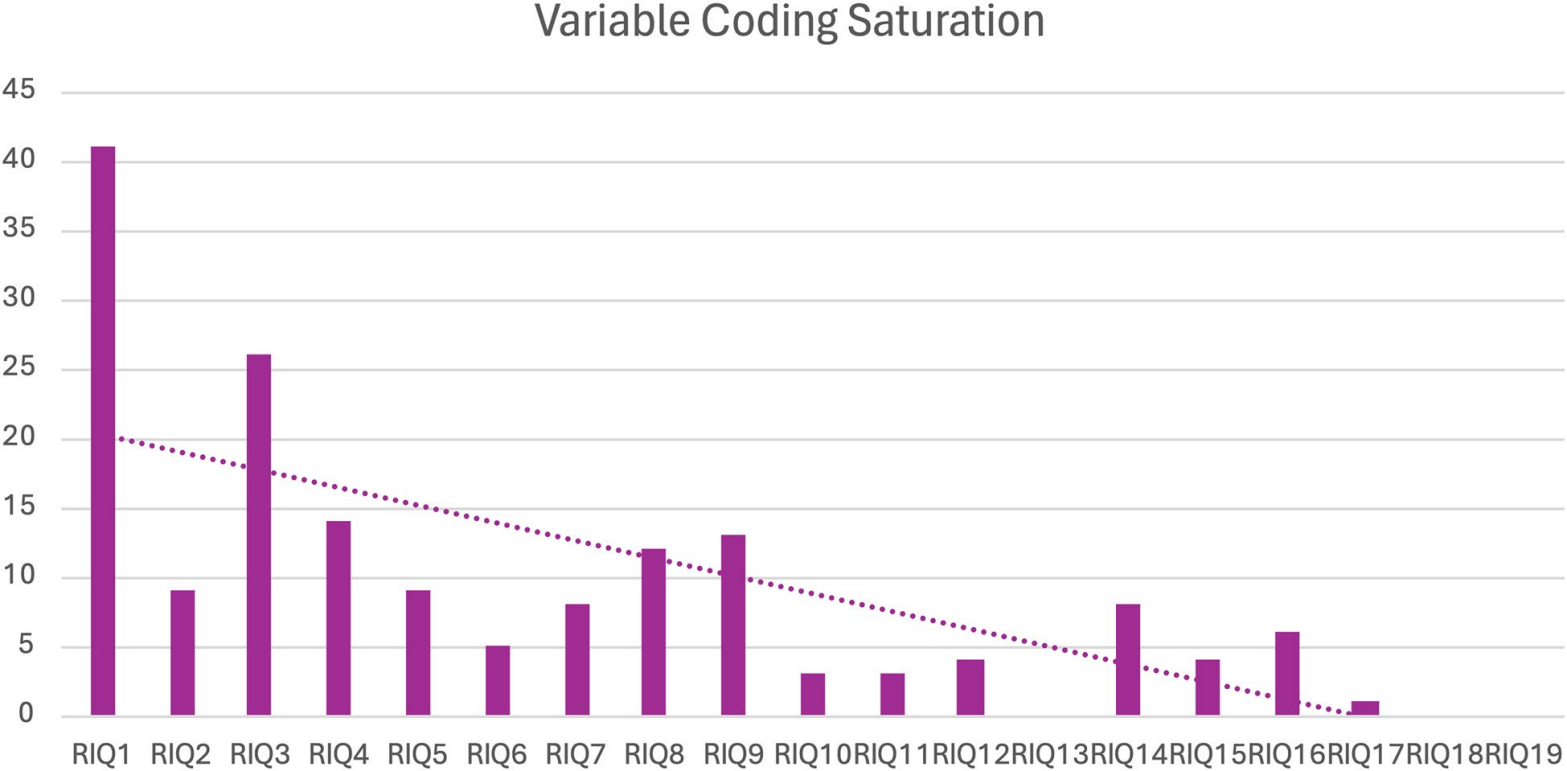
Rigorously interpreted quotation analysis variable saturation

I conducted rigorously interpreted quotation analysis on 19 selected theoretical works, which resulted in 167 unique elements and 338 described causal links. I documented the emergence of new variables at the end of each document to assess for variable saturation. At the end of the 19 originally selected documents, I considered saturation to have been achieved, so I did not seek out any additional references for analysis. Figure 3 shows the decreasing number of new variables obtained in each additional coded document.

Through iterative rounds of causal loop diagram mapping, variable aggregation and causal loop diagram pruning, I was able to distil the variables into 81 unique elements and simplified connections. The connections presented here are drawn from the core body of work related to Kingdon’s multiple streams framework and thus may not entirely represent what would happen in a real system.

**Fig. 4:**
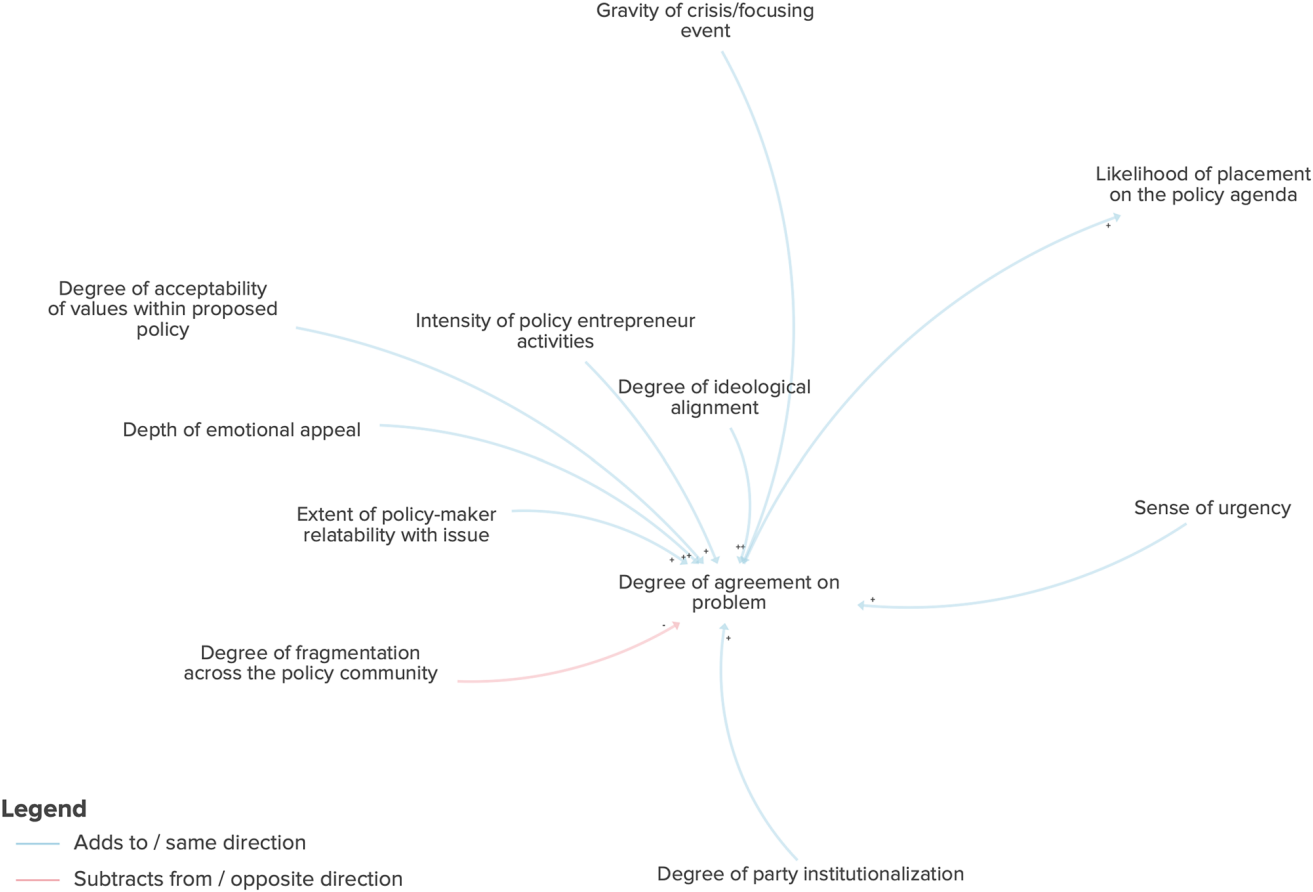
Factors directly influencing the degree of agreement on a problem

I will begin by presenting one of the most prominent variables at the centre of the causal loop diagram: the degree of agreement on problem (Fig. 4). The degree of agreement on the problem is the natural start to the causal loop diagram since it is by recognizing that there is a problem to begin with that the need for a policy solution emerges. According to the rigorously interpreted quotation analysis of the theoretical literature on the multiple streams framework, the key factors that influence the degree of agreement there is on a given problem correspond to four main areas: government characteristics, the policy community, policy-maker characteristics and external events. In terms of external events, an important contributor to the multiple streams framework system is, first, the presence and, secondly, the gravity of a crisis or focusing event. The greater the gravity of this focusing event, the more likely policy-makers will come together and agree on a problem. In terms of policy-maker characteristics, the degree of relatability the policy-maker has with the issue at hand, the depth of the emotional appeal and the acceptability of the values for the corresponding policy solution will increase the degree of agreement on the problem. Additionally, the higher the urgency created by this focusing event, the higher the likelihood of agreement on the problem. Within the policy community, the higher the degree of fragmentation within the policy community, the lower the level of agreement on the problem; however, the intensity of the policy entrepreneur’s activities will increase the degree of agreement. The government characteristics that influence problem agreement include ideological alignment and degree of party institutionalization. Party institutionalization is the degree of stability of a political party and whether it behaves as expected. The higher the alignment of the party’s ideological values with the framing of the problem, the higher the level of agreement. In terms of institutionalization, the more institutionalized a party is, the clearer internal values it will have, and it will generate more ease of agreement on problems.

The degree of agreement on the problem in turn influences another key variable in the system: the likelihood of placement on the policy agenda. And the higher the degree of agreement on the problem, the higher the likelihood the issue will make its way on to the policy agenda.

**Fig. 5:**
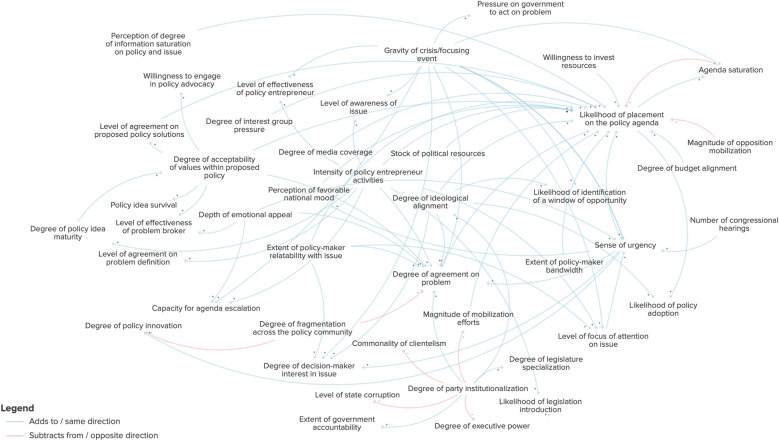
Factors indirectly influencing the degree of agreement on a problem

If we take a step back and look at the indirect connections for the degree of agreement on the problem, we can see how this variable has reach across the multiple streams framework system (Fig. 5). In this expanded view we see that party institutionalization impacts the degree of ideological alignment and that party institutionalization also influences other variables, such as the likelihood of legislation introduction, degree of legislative specialization, extent of executive power, clientelism (the exchange of goods for political support or vote-buying), corruption and government accountability.

If we shift our attention to the likelihood of placement on the policy agenda, we can see a large number of variables that positively influence this and two variables that negatively influence this: magnitude of opposition mobilization and agenda saturation.

**Fig. 6:**
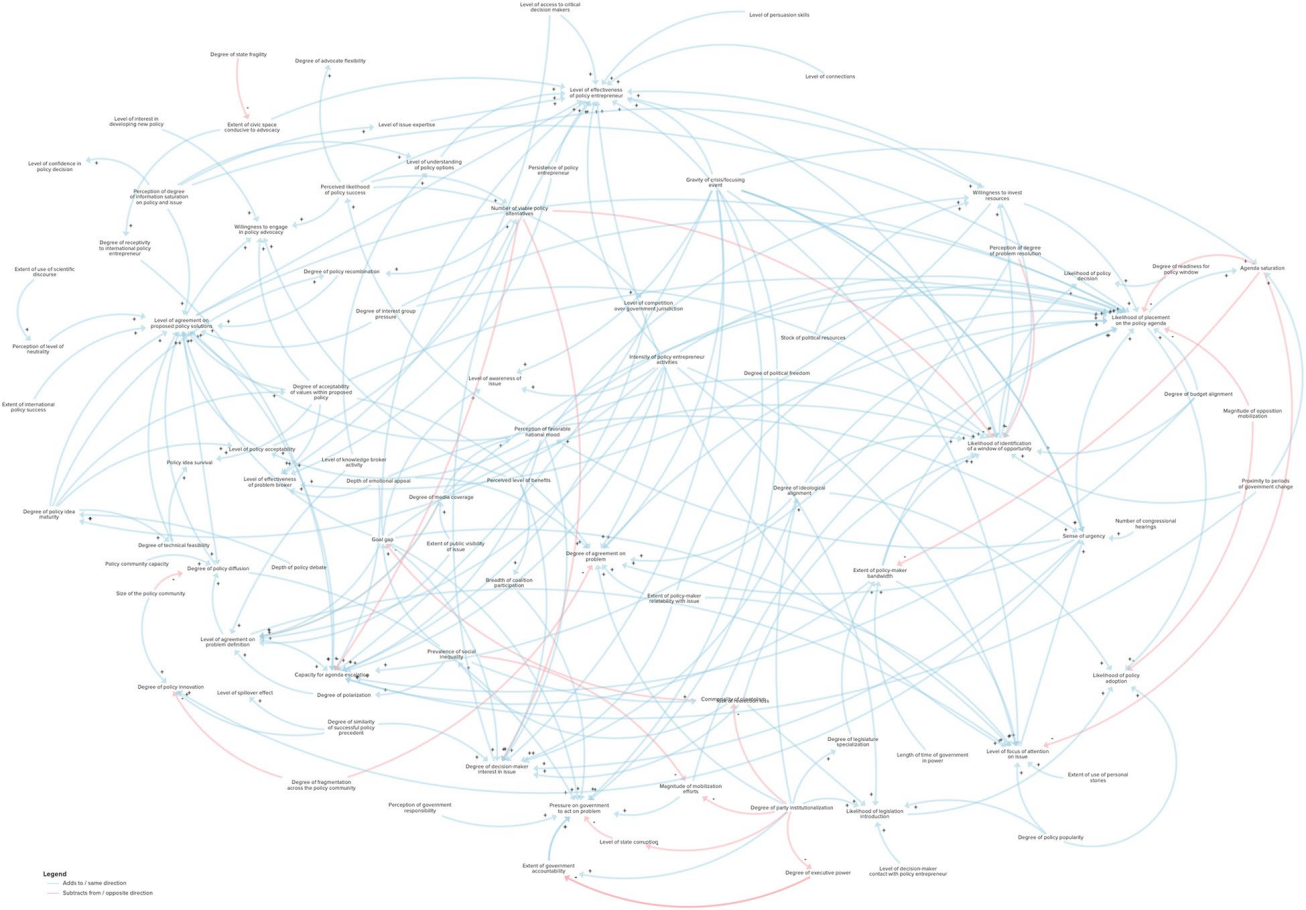
Kingdon’s multiple streams framework causal loop diagram (the diagram can be explored online on Kumu: https://galvarado.kumu.io/kingdons-multiple-streams-framework)

Instead of describing the full causal loop diagram in detail, I will present the most salient balancing and reinforcing loops that emerged in the causal loop diagram and contribute to maintaining the system for agenda setting and policy-making. I summarize the loops and their components in Table 3. The complete aggregated causal loop diagram is presented in Fig. 6.

**Table 3:** Balancing and reinforcing loops present in the multiple streams framework causal loop diagram

Loop name	Loop type	Elements	Diagram	Description
Agenda saturation	Balancing	- Agenda saturation - Likelihood of placement on policy agenda		The less saturated the agenda is, the more likely the issue will make it on to the agenda, which will in turn saturate it for future policy problems.
Social inequality	Reinforcing	- Prevalence of social inequality - Commonality of clientelism		The higher the level of social inequality, the higher the level of clientelism, which in turn increases the prevalence of social inequality.
Policy agreement–diffusion	Reinforcing	- Level of agreement on proposed policy solutions - Degree of policy diffusion		The higher the level of agreement on proposed policy solutions, the higher the degree of policy diffusion, which in turn further increases the level of agreement on the proposed policy solution.
Sense of urgency	Balancing	- Sense of urgency - Likelihood of placement on policy agenda - Agenda saturation - Level of focus of attention on issue		The higher the sense of urgency regarding a particular issue, the more likely it will be placed on the policy agenda. In turn, this will increase the saturation of the agenda, which will decrease the level of focus of attention on the issue. And the lower the level of focus of attention on the issue, the lower the sense of urgency, thus restoring balance to the loop.
National mood–perception of success	Balancing	- Perceived likelihood of policy success - Willingness to invest resources - Perception of favourable national mood - Likelihood of placement on policy agenda - Agenda saturation - Level of focus of attention on issue - Sense of urgency		The higher the level of focus on an issue, the higher the sense of urgency. The higher the sense of urgency, the more favourable the national mood will be for a particular solution. The more perceptible the national mood, the higher perceived likelihood of policy success. The higher the likelihood of policy success, the more resources in terms of time and effort a policy-maker or policy entrepreneur may be willing to make. The more resources invested, the higher the likelihood of placement on the policy agenda, which in turn saturates the policy agenda and then balances out by reducing the level of focus of attention on the issue.
Policy-maker bandwidth–interest	Balancing	- Agenda saturation - Extent of policy-maker bandwidth - Level of focus of attention on issue - Sense of urgency - Perception of favourable national mood - Degree of decision-maker interest in issue - Level of effectiveness of policy entrepreneur - Likelihood of identification of a window of opportunity - Willingness to invest resources - Likelihood of placement on the policy agenda		The lower the level of agenda saturation, the higher the policy-maker’s bandwidth will be to take on work. The higher the bandwidth, the higher the focus of attention on the issue. The higher the focus of attention on the issue, the higher the sense of urgency, which in turn generates the perception of a favourable national mood. The favourable national mood elevates the degree of interest from the decision-maker, which in turn increases the effectiveness of the policy entrepreneur. The more effective the policy entrepreneur, the more likely they are to identify a window of opportunity. When they identify a window of opportunity, they are in turn more inclined to invest more resources in the issue, which increases the likelihood of placement on the agenda. By placing the issue on the agenda, it eventually becomes saturated and balances the loop out.

### Mini case studies

To further assess the appropriateness of the model, I tested it against two case studies on abortion policy reform: Ireland and Nicaragua. I selected these two countries because they represent diverse characteristics such as geographic location, type of reform and mechanism for reform, yet both have strong Catholic traditions. In Ireland, abortion was liberalized through a popular referendum in 2018, while in Nicaragua abortion was further restricted to a complete ban on abortion in 2006 by the country’s legislature amid a contested presidential debate [20]. Drawing from the findings of a metaethnographic review of the abortion policy literature [21], I mapped elements from the published literature on to the proposed causal loop diagram to validate appropriateness of elements, feedback loops and polarity.

#### Ireland

Ireland and Poland are considered to be the most conservative Catholic countries in Europe [22]. In Ireland the Catholic Church was part of the resistance against foreign rule and oppression and as such was very intertwined with sentiments of national identity and held significant societal power [23].

In 1983 Ireland voted in a referendum to establish the Eighth Amendment of the Constitution to ensure “the right to life of the unborn… with due regard to the equal right of the mother”, which reinforced the existing criminalization and bans in access to abortion [24]. This is often cited as having happened in response to the Roe v. Wade ruling in the United States and the Catholic church trying to avoid a potential Supreme Court ruling that the right to marital privacy could include abortion [25].

Before the 2018 repeal via referendum of the Eighth Amendment, public discussion in Ireland surrounding abortion was already changing. In 1992 the case of a 14-year-old girl who was refused the right to travel to England for an abortion led to three new amendments being upheld and travel to England for abortions allowed [24].

In the case of Ireland, the case study literature emphasizes the importance of three factors: a coordinated mobilization effort, decreasing influence of the Catholic church, and having “a face for the movement” [22]. In terms of the causal loop diagram, this can be translated into the magnitude of mobilization efforts and the gravity of focusing event. To zone in on the focusing event, in Ireland there were many focusing events in the form of individuals who had encountered personal difficulties due to the restrictive abortion laws. One case that resonated with the public was the case of Savita Halappanavar, who passed away from complications due to improper management of a miscarriage [26]. The gravity of this event increased the level of awareness of the issues related to restrictive abortion policies but also enhanced the effectiveness of the policy entrepreneur by giving them tools for advocacy and justifications for the policy solution [27–29]. Other closely related variables included the depth of the emotional appeal, the sense of urgency, degree of media coverage and perception of national mood. All of these together create an environment where the decision-maker considers there to be enough interest and likelihood of policy success. The degree of decision-maker interest is also influenced by risks of re-election losses. In the case of Ireland, in the lead up to the 2016 elections, many political parties noticed how the national mood was shifting and how abortion policy was a priority to many people, and thus used the issue of abortion to capture a segment of votes. These parties then committed themselves to holding a referendum to repeal the Eighth Amendment and liberalize abortion [29].

#### Nicaragua

Nicaragua, the largest country in Central America, is one of the poorest in the Latin American region, second only to Haiti [30]. Daniel Ortega, the leader of the Sandinista leftist party, has been the president since 2006 and, prior to the 2006 elections, supported legalized abortions [31]. According to census data from 2005, around the time of the 2006 presidential elections more than 80% of the population identified as part of a Christian religion, of which the majority (70%) were Catholic [32]. This is why, after losing elections in 1990, 1996 and 2001, Ortega sought the support of the Catholic church [31]. Leading up to the election he touted himself as being “more Christian than Marxist” nowadays and voiced support for abortion policy reform to further restrict an already restrictive law that had been in place for 130 years and only allowed for therapeutic abortions [33].

On 26 October, just 5 days before the 2006 presidential elections, the Nicaraguan legislature passed a complete ban on abortion when the leftist Sandinista block all voted in favour of the removal of the penal code provision that allowed for therapeutic abortions for three specific instances: (1) to save the life of the mother, (2) in cases of rape or incest or (3) when the fetus had severe malformations [34–36]. The provision passed unanimously despite pleas from physicians, Ministry of Health officials, international human rights entities and feminist groups to postpone the vote until after the election [34]. The change in abortion policy was broadly viewed as a political deal that was struck before the election to ensure the votes the Sandinista party needed to return to power [34, 37]. Nicaragua is now one of 21 countries in the world that maintain a complete abortion ban, and one of four countries that has rolled back abortion rights in lieu of more severe restrictions [2, 38].

Prior to 2005, efforts for abortion reform had been made between 1999 and 2001. In 1999 discussions began regarding an abortion policy amid a broader Penal Code reform, which would have aimed to explicitly include mentions of rape, fetal malformations and health of the mother in the Penal Code, as well as the requirement for three medical specialists to sign off on the procedure, consent from a family member, and lastly a new article that punished harm to the unborn [39]. Despite the fact that the proposed reform would have significantly reduced access to abortion, religious groups were strongly opposed and argued for the complete elimination of therapeutic abortion from the Penal Code, while feminist groups argued that these reforms would severely impact access. By 2001, as the presidential elections drew near, the Penal Code reform was postponed and remained that way until the 2005 abortion ban came into effect [39].

Nicaragua is not the only country in which political parties have made campaign promises that fall flat. However, when we test this case against the developed MSF causal loop diagram, we can see that the change in position on abortion was due to the degree of party institutionalization. Unlike Ireland, where the parties that committed themselves to advancing abortion policy on the policy agenda were well institutionalized, meaning that the political party was stable and political actors behaved in stable and expected ways, the political party led by Ortega in the 2006 elections was not well institutionalized [40, 41]. In the causal loop diagram, we can see that the degree of institutionalization is associated with the likelihood of actual placement on the policy agenda and/or introduction of legislation. As expected, given the low level of party institutionalization in Nicaragua, Ortega soon created an alliance with the Catholic Church to increase voter shares and turned his stance to a firmly anti-abortion one instead [41]. This is how, instead of liberalizing the abortion policy, Nicaragua increased restrictions and removed “saving the life of the pregnant person” from grounds to allow for abortion. Since then, abortion is strictly prohibited under all circumstances. In Nicaragua there was also a highly publicized case where a 9-year-old child known to the media as “Rosita” sought an abortion after being the victim of sexual abuse and becoming pregnant [42]. This case could have paralleled Savita’s in Ireland to constitute a focusing event; however, due to the government characteristics in Nicaragua, advocacy efforts were not effective. These government characteristics are depicted in the causal loop diagram and include clientelism, corruption and high degree of presidentialism (concentration of power within the executive).

## Discussion

In this study I conducted a review of the most influential works on Kingdon’s MSF and used a systems dynamics approach to conduct rigorously interpreted quotation analysis and aggregated the resulting data into a causal loop diagram on Kumu. Beneath the parsimonious MSF, I was able to ascertain that the streams are reflective of the complex dynamics of agenda setting, and more specifically abortion policy agenda setting [6, 43]. Furthermore, the causal loop diagram shows numerous interconnections between the three original MSF streams, which may indicate that convergence of the streams into windows of opportunity is due to more than just chance and randomness.

In the “Results” section I presented first the end goal of the model, which is getting an issue placed on the policy agenda. However, this causal loop diagram allows us to back-trace all the conditions and interactions necessary to generate an outcome with high likelihood of placement on the policy agenda. In reviewing the types of variables and interactions, I found four broad types of variables that influence the system: (1) government characteristics, (2) the policy community, (3) policy-maker characteristics and (4) external events.

Kingdon often describes the MSF as a game of wait and see, where policy entrepreneurs are like surfers “waiting for the big wave” and prepare for that optimal window [44]. However, in disaggregating the MSF and visualizing through a causal loop diagram, we can see that there are many points where the system can be influenced to increase the likelihood of said windows of opportunity. These points are commonly referred to as leverage points, where a small change in an element can lead to observable effects in the system [45].

Donella Meadows proposed a list of places where one can typically intervene in a system [45], and several of these match critical points in the MSF causal loop diagram I developed, as shown in Table 4. Meadows’ list of places to intervene include the goals of the system, the paradigm out of which the system operates, the distribution of power over the rules of the system, information flows, material flows and nodes of intersection, the driving positive feedback loops, the regulating negative feedback loops and constants and numbers. To translate these into the multiple streams framework, we can see the goals of the system as the framing of the problem or, as it is represented in the causal loop diagram, the degree of agreement on the problem. In terms of the feedback loops, two feedback loops that emerged in the development of the causal loop diagram that regulate the MSF include the social inequality loop and the agenda saturation loop (both depicted and described in Table 3). Intervening in the social inequality loop would mean that, by reducing social inequality, you would reduce the prevalence of clientelism. This reduction in clientelism in turn increases the overall accountability and transparency of the government, which means that it is more responsive to pressure from mobilization efforts. The agenda saturation loop speaks to the general bandwidth of decision-makers and policy-makers. When agendas are highly saturated, this means there is a low likelihood there is room to add on additional issues. This is a critical place to intervene in terms of expanding the capacity of the system. Elements that are related to the degree of agenda saturation include degree of party institutionalization, legislature specialization, ideological alignment, length of time of the current government in power and gravity of focusing events.

**Table 4:** Places to intervene within a system – overlap between Meadows’ list and findings from the multiple streams framework causal loop diagram analysis

Meadows’ original list	Multiple streams framework equivalent
Goals of the system	Framing of the problem, or the degree of agreement on the problem
Distribution of power over the rules of the system	Degree of presidentialism (executive power)
Positive feedback loops	Social inequality loop
Negative feedback loops	Agenda saturation loop
Constants and numbers	Indicators or goal gaps

Meadows’ distribution of power matches the concept of degree of executive power, or how much power is concentrated in the president; the information flows can be paralleled by the degree of fragmentation of the policy and scientific communities; and lastly the constants and numbers are the indicators or goal gaps that policy-makers care about and that create a sense of urgency. When this goal gap increases, we become aware that there is an issue, more attention is given to the issue and, eventually, there is a higher likelihood of placing on the policy agenda. If there is no goal gap to point to, there is no leverage for change, because “if it ain’t broke, why fix it”.

In the case of abortion policy, one of these goal gaps that is constantly referenced in the academic literature is the level of maternal mortality [20, 46–49]. Rises in reported maternal mortality have the capacity to create widespread awareness that there is an issue if they are leveraged accordingly by a policy entrepreneur. The goal gap also contributes to the policy community coalescing around a proposed solution and increasing agreement on how to solve the issue. The goal gap also empowers the policy entrepreneur act on the issue. All of these contribute to placement on the policy agenda. However, rises in maternal mortality do not always lead to abortion policy reforms. The causal loop diagram helps us understand all the other mediating elements that can explain this apparent discrepancy. For example, a central set of elements in the causal loop diagram are comprised by policy entrepreneur actions. If there is no interested actor to change the status quo, the system will not budge. Furthermore, the actions of the policy entrepreneur are also limited by other variables of the system; including the degree of power and authority of the policy entrepreneur themselves; the level of polarization of the issue; degree of ideological alignment between the problem, proposed solution and ruling party; and the overall receptivity of the government to mobilization and lobbying efforts.

At the same time the goal gap is driven by inequality, which is driven by ineffective governments. Governments with a low level of party institutionalization, high corruption and high clientelism are ineffective and inadvertently increase inequality. These inequalities are reflected in the goal gap. However, the government characteristics will determine how the system responds to this goal gap. In a highly effective and democratic country with government accountability and transparency, there is an environment that is conducive for civil society engagement, and elected officials respond to public pressure since they risk losing re-elections. If the type of government is not conducive to advocacy efforts, then policy reform will be very unlikely unless it is in the personal interest of an individual with high political power. This is why I have identified as important leverage points the democratic and political structures of the government.

The causal loop diagram shows that a party with low levels of institutionalization will have poor ideological alignment and will not be able to come to agreement on a problem definition nor a policy solution. Levels of institutionalization also impact the degree of specialization of the legislature, the degree of executive power and likelihood of legislation introduction, all of which determine the conduciveness of the policy advocacy space. Low levels of institutionalization are associated with increased clientelism, which further widens social inequality.

Lastly, many of the connections in the causal loop diagram are linked to the effectiveness of the policy entrepreneur. This model shows that, without an actor with a stake in the issue and steering the ship, issues would take much longer to get on to the policy agenda if they are not a specific priority to policy-makers. Systems thinking allows us to understand these internal forces that exist within the system to then mobilize them in our favour and create the outcomes that we are interested in [50]. I believe that critical points that are important for policy-mobilization are highlighting the goal gap and ensuring that there is a responsive political structure in place.

In the case study literature, there are documented instances of broad coalitions and mobilization efforts for the liberalization of abortion policy. However, it may be that the system is not yet prepared to be responsive to advocacy efforts from a policy entrepreneur, and instead the movement might benefit from coalitions with political engagement and voter education approaches.

While the causal loop diagram is promising, this is exploratory work with opportunities for improvement. Limitations of the work include that the text data used for this analysis came from published academic articles and, as such, is not purposive. It was not written with the aim of establishing causality between elements; hence, some of the connections and associations are lax, and other connections which may seem obvious from the outside are absent. However, this was mitigated by establishing a clear codebook with specific coding rules that could be replicated and increase reliability [16]. Furthermore, the causal loop diagram developed here may serve as foundational data to supplement with purposive interviews designed to elicit causal language.

Another limitation is that the causal loop diagram is a closed feedback system – this means it can only account for changes in endogenous variables [51]. This means that it is important to capture the behaviour of interest within the system boundary, and this may not be possible with a small subset of academic papers.

## Conclusions and theoretical implications

This study set out to address a critical gap in the abortion policy literature: understanding how abortion emerges on the policy agenda, rather than focusing solely on policy outcomes or debates. By integrating Kingdon’s MSF with systems thinking and causal loop diagramming, this research demonstrates that the MSF, despite its parsimony, is capable of capturing the complex, dynamic and interdependent factors that shape agenda setting in contentious policy domains such as abortion.

The causal loop diagram developed here reveals that the convergence of the problem, policy and politics streams is not merely a matter of chance but rather is influenced by a web of reinforcing and balancing feedbacks among government characteristics, policy communities, policy-maker attributes and external events. The case studies of Ireland and Nicaragua illustrate how these dynamics play out in practice, highlighting the importance of factors such as party institutionalization, policy entrepreneur effectiveness and the gravity of focusing events in shaping policy trajectories.

Furthermore, this approach identifies actionable leverage points – such as the degree of party institutionalization, the effectiveness of policy entrepreneurs and the management of social inequality, which can inform advocacy strategies and policy interventions. The model also provides a foundation for future empirical research, including qualitative interviews and expanded case studies, to further validate and refine the framework.

In sum, this study bridges theoretical innovation with practical relevance, offering new tools and perspectives for scholars, advocates and decision-makers seeking to understand and influence the policy agenda for abortion and other complex health issues.

## Appendix

Which works from the literature on Kingdon’s multiple streams framework were used for rigorously interpreted quotation analysis for the causal loop diagram?

This table provides a brief summary of each of the 19 included references used to develop the causal loop diagram.

See Table 5

**Table 5:** Key relevant points from each included reference

Reference	Key relevant points
Anderson, S.E., DeLeo, R.A., Taylor, K. (2019)[52]	This article assesses the interactions between policy entrepreneurs and legislators and the implications for agenda setting and policy change.
Béland, D. [7]	This paper explores the contributions of the multiple streams framework to the field of comparative policy analysis, in particular how the three streams relate to the role of institutions and the relationship between ideas and interests.
Blum, S. (2017)[53]	This article brings together theories of knowledge utilization in the operationalization of multiple streams framework’s stream coupling by developing a concept called argumentative coupling where the policy, problem and politics streams are linked by arguments.
Cairney, P., Jones, M.D. [43]	This article provides relevant context on the multiple streams framework and provides a broad assessment of how the multiple streams framework has contributed to the political science space. The authors propose that the main contributions include providing a baseline for other theories to be developed such as punctuated equilibrium, and the growth of a dedicated empirical literature.
Fowler, L. (2018) [54]	This study explores the applicability of the multiple streams framework beyond policy change to policy implementation.
Herweg, N., Huß, C., Zohlnhöfer, R. (2015) [55]	The multiple streams framework was created with the US federal system in mind. This article explores ways to refine and expand the model to be used in parliamentary systems and to understand the decision-making process after the issue has been placed on the policy agenda.
Herweg, N., Zahariadis, N., Zohlnhöfer, R. (2017) [56]	This book chapter summarizes the state of the multiple streams framework literature, theoretical refinements, innovations and empirical applications. The chapter also expands on the uses of the multiple streams framework beyond agenda setting to decision-making and policy implementation.
Howlett, M., McConnell, A., Perl, A. (2014) [57]	This article examines opportunities to combine the multiple streams framework with the policy process steps of policy formation and decision-making to add nuance and complexity to the model, as well as increasing its analytical value.
Jones, M.D., Peterson, H.L., Pierce, J.J., Herweg, N., Bernal, A., Lamberta Raney, H., Zahariadis, N. (2015) [58]	This meta-review examines 311 publications that have applied elements of the multiple streams framework within their analysis and aggregate through content analysis how authors operationalize elements from the multiple streams framework in their work.
Kingdon, J.W. (2011a) [59]	This chapter from the book *Agendas, Alternatives and Public Policies*, provides an in-depth description of the various components within the problem stream.
Kingdon, J.W. (2011b) [60]	This chapter from the book *Agendas, Alternatives and Public Policies*, provides an in-depth description of the various components within the policy stream.
Kingdon, J.W. (2011c) [61]	This chapter from the book *Agendas, Alternatives and Public Policies*, provides an in-depth description of the various components within the politics stream.
Kingdon, J.W. (2011d) [62]	This chapter from the book *Agendas, Alternatives and Public Policies*, details the joining of the three streams to form a policy window
Knaggård, Å. (2015) [63]	This paper discusses the advancement of the multiple streams framework through the inclusion of a distinct policy broker who works to frame issues as public problems and convincing policy-makers that these issues are indeed problems. The policy entrepreneur then leverages the work of the policy broker to create windows of opportunity.
Rawat, P., Morris, J.C. [6]	This article provides a comprehensive review of the academic literature related to the multiple streams framework. The authors report most common uses of the multiple streams framework and recommend areas for future exploration.
Sanjurjo, D. (2020) [64]	This article examines how the multiple streams framework can be exported to Latin American contexts and suggests that in order for the multiple streams framework to be applicable it needs to take into consideration the possibility of weak parties, external powerful actors and corruption.
Shepard, D.D., Ellersiek, A., Meuer, J., Rupietta, C., Mayne, R., Cairney, P. (2020) [65]	This qualitative comparative analysis explores the applicability of the multiple streams framework across different aid-receiving nations and notes the limitations of the multiple streams framework. One of the main limitations noted by the authors is the assumption of a liberal democracy and the freedom for civic engagement and policy entrepreneurs to effectively engage in the policy-making process. Furthermore, they add an additional element of a global policy entrepreneur.
Zahariadis, N. (2014) [66]	This study explores the role of emotion within the multiple streams framework and how different types of emotions can have an impact on policy change.
Zohlnhöfer, R., Herweg, N., Rüb, F. (2015) [67]	This is the introductory article of a special edition of the *European Journal of Political Research* that focuses on theoretical refinements and debates surrounding the multiple streams framework.

## Data Availability

No datasets were generated or analysed during the current study.

## Abbreviation

MSF: Kingdon’s multiple streams framework

## Footnotes

1 https://www.dedoose.com

2 https://kumu.io.
